# Zn- or Cu-Containing CaP-Based Coatings Formed by Micro-arc Oxidation on Titanium and Ti-40Nb Alloy: Part I—Microstructure, Composition and Properties

**DOI:** 10.3390/ma13184116

**Published:** 2020-09-16

**Authors:** Ekaterina G. Komarova, Yurii P. Sharkeev, Mariya B. Sedelnikova, Konstantin A. Prosolov, Igor A. Khlusov, Oleg Prymak, Matthias Epple

**Affiliations:** 1 Laboratory of Physics of Nanostructured Biocomposites, Institute of Strength Physics and Materials Science SB RAS, 634055 Tomsk, Russia; sharkeev@ispms.tsc.ru (Y.P.S.); smasha5@yandex.ru (M.B.S.); konstprosolov@gmail.com (K.A.P.); 2 Research School of High-Energy Physics, National Research Tomsk Polytechnic University, 634050 Tomsk, Russia; 3 Department of Morphology and General Pathology, Siberian State Medical University, 634050 Tomsk, Russia; khlusov63@mail.ru; 4 Research School of Chemistry and Applied Biomedical Sciences, National Research Tomsk Polytechnic University, 634050 Tomsk, Russia; 5 Inorganic Chemistry and Center for Nanointegration Duisburg-Essen (CeNIDE), University of Duisburg-Essen, 45141 Essen, Germany; oleg.prymak@uni-due.de (O.P.); matthias.epple@uni-due.de (M.E.)

**Keywords:** micro-arc oxidation, calcium phosphate coating, pure titanium, Ti-40 wt% Nb alloy, microstructure, morphology, adhesion strength

## Abstract

Zn- and Cu-containing CaP-based coatings, obtained by micro-arc oxidation process, were deposited on substrates made of pure titanium (Ti) and novel Ti-40Nb alloy. The microstructure, phase, and elemental composition, as well as physicochemical and mechanical properties, were examined for unmodified CaP and Zn- or Cu-containing CaP coatings, in relation to the applied voltage that was varied in the range from 200 to 350 V. The unmodified CaP coatings on both types of substrates had mainly an amorphous microstructure with a minimal content of the CaHPO_4_ phase for all applied voltages. The CaP coatings modified with Zn or Cu had a range from amorphous to nano- and microcrystalline structure that contained micro-sized CaHPO_4_ and Ca(H_2_PO_4_)_2_·H_2_O phases, as well as nano-sized β-Ca_2_P_2_O_7_, CaHPO_4_, TiO_2_, and Nb_2_O_5_ phases. The crystallinity of the formed coatings increased in the following order: CaP/TiNb < Zn-CaP/TiNb < Cu-CaP/TiNb < CaP/Ti < Zn-CaP/Ti < Cu-CaP/Ti. The increase in the applied voltage led to a linear increase in thickness, roughness, and porosity of all types of coatings, unlike adhesive strength that was inversely proportional to an increase in the applied voltage. The increase in the applied voltage did not affect the Zn or Cu concentration (~0.4 at%), but led to an increase in the Ca/P atomic ratio from 0.3 to 0.7.

## 1. Introduction

During the most recent couple of decades, an outstanding amount of studies, advancing the area of biocompatible material production, has been conducted [1,2]. An ever-increasing number of cutting-edge biomedical innovations, materials, and items are being created, including metal-based implants and biocompatible coatings, which could replace damaged bones and foster healing. Factors, such as tissues, cellular and biomechanical compatibility are of utmost importance for the functional reliability of such materials.

There are two main approaches for the improvement of medical devices that would increase their performance and service life. The first approach is the development of metallic materials that would be biomechanically compatible with a host tissue preventing possible degenerative processes in bones or even decreasing the risk of revision surgery [1,2,3,4]. The second approach is the development of advanced calcium phosphate (CaP) coatings that could be tailored, in terms of compositional, structural, and morphological features, enhancing the osteogenic potential in the post-operative period [2,5,6,7]. The implant performance is defined by a complex of factors, including biological, physical, chemical, and mechanical effects. These factors impose high requirements for the biological and mechanical compatibility of implant materials. However, most biocompatible metals nowadays lack the required properties. This is evident through a significant number of unsuccessful implantation outcomes, including implant failure [1,2,3,4].

The most widely used alloys in medicine are commercially pure titanium (Ti, Grade 2–4), titanium alloys (e.g., Ti-6Al-4V, Ti-6Al-4V ELI) and vanadium-free alloys (e.g., Ti-6Al-7Nb, Ti-6Al-2.5Fe). However, minimizing or eliminating toxic alloying elements is desirable, including Аl, V, Mo, etc., that could negatively affect the organism. In this regard, the most used material for biomedical applications are the bioinert valve metals, such as titanium, zirconium, niobium, hafnium, tantalum, and their alloys [1,2,3,4]. It is important to note that these alloys should not only high strength properties (yield strength, fatigue strength, wear resistance, etc.), but also a low elastic modulus, adapted to that of bone. The elastic modulus is an important functional characteristic of implantable materials. One of the causes of implant failure is a mismatch in elastic modulus between the implantable device and bone, leading to either stress shielding or insufficient mechanical support. Low elastic modulus alloys provide a uniform distribution of strain and stress in the bone-implant interface, thereby, preventing the stress-shielding and increasing the service life of an implant. The elastic modulus of titanium and its alloys ranges from 100 to 120 GPa, which is significantly higher than the elastic modulus of bone tissue (10 to 40 GPa) [3,4]. Accordingly, the most promising research area in material science for medicine is the development and application of binary β-type titanium alloys of Ti-Nb system that have a low elastic modulus [8]. Many researchers [9,10,11] agree that elastic properties mainly depend on the alloy structure, which in turn, depend on the content of the alloying elements. Hon et al. [10] and Lee et al. [11] showed that the alloying of titanium with 40–45 wt% niobium allows to reduce the Young modulus to 55–60 GPa and to stabilize the β-phase with reduction of α′ phases. Although, surface modifications are frequently used to further improve implants performance.

In order to prevent postoperative complications caused by a rejection, it is necessary to deposit coatings on the surface of implants. Such coatings should be biologically active and biocompatible. The surface modification of the metal implants promotes osteogenesis and osteointegration of the bone tissue, as well as improvement in corrosion resistance, creating an effective chemical barrier against ion extraction from the metallic substrate [7,12]. For this purpose, it is most promising to use bioactive CaP-based coatings with a porous structure and developed surface morphology that incorporates the natural biomineral of bone tissue [13].

Over the last years, the micro-arc oxidation (MAO) technique also known as plasma electrolytic oxidation (PEO) has being widely used as an electrochemical and plasma-chemical method for the formation of oxide ceramics and CaP-based coatings on the surface of valve metals, e.g., Al, Ti, Zr, Mg and their alloys [5,7,14,15]. During the MAO procedure, the thermochemical processes occur in the regions of the local micro-arc discharges under the influence of a high voltage generated by a power supply, thereby, resulting in a modified surface of a metal substrate. Generally, the MAO coatings have a broad spectrum of physical and chemical properties, high corrosion and wear resistance, high micro-hardness and adhesion strengths [16,17,18]. The structure, composition, and properties of the coatings are determined by the MAO parameters, such as the electrolyte composition, the elemental composition of substrate material, electrical voltage, current density, time, etc. Most scientists [18,19] use the true electrolytic solutions or electrolytic suspensions that are incorporated with stoichiometric hydroxyapatite (HA) nano-powder that enhances biocompatibility and bioactive properties, as well as increase corrosion resistance of titanium substrates and its alloys. While, incorporating Ca and P is common, the addition of modifying additives, such as Ag, Sr, Zn or Cu into the coating composition is possible by adding it in an electrolyte of ion substituted hydroxyapatites, as shown in our previous reports [20,21,22]. 

One of the main challenges in biomedicine is a bacterial infection of medical implants. Bacterial infection is a result of bacteria adhesion to the implant surface, accompanied by a production of the extracellular matrix, leading to biofilm formation that can be difficult to treat with antibiotics [23]. Therefore, the aim of many research groups is on the synthesis of biomaterials with antibacterial properties. The thorough investigation of the activity and interaction patterns of antibacterial agents with surrounding tissues is of great interest to the biomedical field [24,25]. The development of inorganic antibacterial biomaterials with high antibacterial activity, biosafety, and osteoconductivity is of vital importance. Many research groups [26,27,28] suppose that the antibacterial and anti-inflammatory effect of implanted materials is mainly due to the incorporation in their structure-specific elements that possess natural bactericidal properties. In the recent reports [26,29,30,31,32] it was shown that zinc (Zn) and copper (Cu) ions, and in particular, possess substantial antimicrobial activity against various bacteria (e.g., gram-positive *S. aureus* and *S. epidermidis*; gram-negative *E. coli* and *P. aeruginosa*). It is worth mentioning that Zn and Cu are vital cofactors influence the enzymes involved in the synthesis of various components of the bone matrix, and are especially essential in governing bone resorption and sedimentation. Additionally, Zn is a crucial microelement of the human body. It plays an important role in various biological functions, such as hormonal activity, the activity of enzymes, DNA synthesis, metabolism of nucleic acids, and biomineralization [33]. It has been shown that Cu not only possesses antibacterial properties, but its deficiency can cause disorders in the skeletal system, hence, Cu is vitally important for living organisms [32,33]. Therefore, the incorporation of Zn or Cu modifying additives into biocoatings could promote the direct antimicrobial activity and prevent the growth of pathogenic microorganisms. We also suppose that the Zn or Cu incorporation into the coatings can balance microelement concentrations between the biocoatings and bone tissue, as well as improve the biological conditions for the implant adaptation to the surrounding environment.

However, despite a sufficient amount of reports on the deposition of CaP coatings modified by Zn and Cu elements, there is still no general consensus on how the structural, morphological and physicochemical properties and elemental composition of the coatings correlate with the release of doping elements and the biological and microbiological activity of the coatings. In addition, the amount of available research relating to modified CaP coatings on a novel titanium-niobium alloy with low elastic modulus is very limited.

Therefore, the present work aims to describe the synthesis of the CaP-based (CaP), Zn-containing CaP (Zn-CaP), and Cu-containing CaP (Cu-CaP) coatings by the MAO method on Ti and Ti-40 wt% Nb alloy and subsequent study of the coatings′ microstructure, phase, and elemental compositions, their physicochemical and mechanical properties.

## 2. Materials and Methods

### 2.1. Preparation Procedures and MAO Treatment

The samples used in this study were cut from billets of commercial pure titanium (Grade 2, VSMPO-AVISMA Corp., Verkhnaya Salda, Russia) and Ti-40 wt% Nb (Ti-40Nb) alloy in the form of plates of 10 × 10 × 1 mm^3^ in size. The β-phase Ti-40Nb alloy was produced using an electron arc melting method with a non-consumable electrode (General Research Institute for Nonferrous Metals, Beijing, China) [34,35]. The specimens were ground in series of increasingly abrasive paper grits up to P1200 using the polishing machine (Tegra System, Struers, Denmark). Then, the samples were ultrasonically cleaned (Elmasonic S, Elma, Germany) in distilled water and ethanol for 10 min and dried in air.

To synthesize the MAO coatings, three types of the electrolyte containing 27 wt% H_3_PO_4_, 7 wt% CaCO_3_, 5 wt% nano-sized hydroxyapatite (HA) and distilled water as the balance were taken. Firstly, to prepare the CaP coatings, the stoichiometric HA nano-powder (Ca_10_(PO_4_)_6_(OH)_2_) was used. For the Zn-CaP coatings, a substitution method of Ca^2+^ by Zn^2+^ cations leading to Zn-substituted HA (Zn-HA, Ca_9.9_Zn_0.1_(PO_4_)_6_(OH)_2_) was applied. Finally, for the Cu-CaP coatings, the Cu-substituted HA (Cu-HA, Ca_9.9_Cu_0.1_(PO_4_)_6_(OH)_2_) within the cationic substitution of Ca^2+^ ions by Cu^2+^ ions were used. The solid-phase mechanochemical synthesis was done to produce all HAs according to the procedure described in the ref. [36,37,38]. All suspension-electrolytes had acid medium with pH of 1–2.

**Figure 1:**
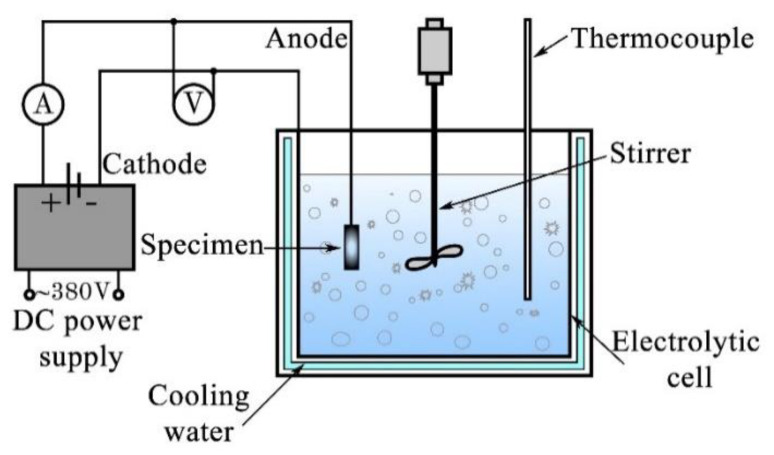
A schematic illustration of the “Micro-Arc 3.0” experimental setup.

The “Micro-Arc 3.0” experimental setup was used to deposit the MAO biocoatings on Ti and Ti-40Nb substrates, as described in detail in our previous reports [20,21,22,39]. Figure 1 shows a schematic representation of the experimental setup, consisting of pulsed direct current (DC) power supply, the bath of electrolyte with the water-cooling system, and counter electrodes. The setting parameters of the coating deposition were controlled by a computer. In our case, the sample and the titanium electrolytic bath played a role of working (anode), and the counter (cathode) electrodes, respectively. During the deposition, a mechanical stirrer continuously mixed the electrolyte to prevent clustering and aggregation of particles in aqueous solution. The thermocouple controlled the temperature of electrolyte keeping it below 30 °С. In this work, the MAO coatings were synthesized using a unipolar anodic potentiostatic regime at a fixed pulse frequency of 50 Hz and a pulse duration of 100 µs for 10 min. The MAO voltage was varied from 200 to 350 V for Ti samples and from 200 to 300 V for Ti-40Nb samples with a step of 50 V (Table 1).

### 2.2. Morphology and Topography Characterization

The surface and cross-sectional morphology, as well as the elemental distribution and composition of the MAO coatings on Ti and Ti-40Nb substrates, was analyzed by scanning electron microscopy (SEM) LEO EVO 50 electron microscope (Zeiss, Oberkochen, Germany). The porosity was calculated using the equation: *P* (%) = Σ*l*/Σ*L* × 100, where *L* is the full length of secants on the SEM images, and *l* is the length of the secants within the pores as described in our previous report [39]. The sizes of structural elements were measured by the secant method, using SEM images, according to ASTM E1382-9 and DD ENV 1071-5 standard protocols. The surface average roughness (*Ra*) was estimated using a profilometer (Hommel-Etamic T1000 basic, Jenoptik, Jena, Germany). The traverse length and speed of the measured profile were 6 mm and 0.5 mm/s, respectively. A universal testing machine (Instron-1185, Great Britain) was used to measure the coating adhesion strength in the pull-off adhesion test regime. Two cylinders were glued to both sides of the coated specimens and were pulled with a stretch rate of 0.1 mm/min at room temperature. The adhesion strength was calculated using the formula: *δA* = *F*/*S*, where *F* is the force at which adhesive or cohesive failure occurred and *S* is the separation area as described in our previous work [39].

### 2.3. Phase, Elemental and Chemical composition

X-ray powder diffraction (XRD) measurements of the coated substrates were carried out on a D8 Advance diffractometer (Bruker, Karlsruhe, Germany) with Bragg-Brentano geometry using Сu Kα radiation (1.54 Å; 40 kV and 40 mA). The flat samples were fixed in the sample holder by a clay dough to keep the right position and measured in the 2*θ* range from 5° to 90° with a step size of 0.01° and a counting time of 1.5 s per step. For the qualitative phase analysis, the software Diffrac Suite EVA V1.2 from Bruker was used taking the patterns of HA (#09-0432) from the International Centre for Diffraction Data (ICDD) database as references. To determine the lattice parameters and crystallite size of detected apatite phases, Rietveld refinement was performed with the program package TOPAS 5.0 from Bruker and realized after the successful instrumental characterization of diffractometer by measuring a standard powder sample LaB6 from NIST (SRM 660b; *a*(LaB_6_) = 4.15689 Å).

The elemental composition and distribution were studied by energy-dispersive X-ray spectroscopy (EDX) using the INCA analyzer (Oxford Instruments, High Wycombe, UK) in combination with the SEM systems. Fourier transformed-infrared spectroscopy (FT-IRS) was performed using Alpha IR-spectrometer (Bruker, Bremen, Germany) in the reflection mode in the wavenumber range of 500–3000 cm^−1^.

### 2.4. Microstructure Characterization

The coatings′ microstructure was investigated by the JEM 2100 transmission electron microscope (TEM) (JEOL, Tokyo, Japan). The accelerating voltage was set to 200 kV, the point and line resolution was 0.19 nm and 0.14 nm, respectively. For the TEM study, the replica with the particles removed from the CaP coating layer was prepared. The analysis of selected area electron diffraction (SAED) patterns was performed using the ICDD PDF 4+ database.

Experimental equipment for SEM, TEM, and EDX studies was provided by the “Nanotech” Common Center for Collective Use (ISPMS SB RAS, Tomsk, Russia).

## 3. Results and Discussion

### 3.1. MAO Treatment

The effects of the pulse voltage magnitude on the structure, elemental composition, and properties of the coatings were studied. In our case, the minimum of the applied voltage for the occurrence of micro-arc was found to be 150 V. However, this regime led to the formation of the thin heterogeneous coating represented by local fragments. The voltage values of 400 V and higher, on the other hand, provided transformation of the micro-arc process to arc oxidation on Ti substrates. This led to the coating’s spark-wearing and subsequent destruction. In the case of the Ti-40Nb substrate, however, the coating was already damaged at the voltage of 350 V and higher. On this basis, the applied voltage values in our experiments were set to 200–350 V for Ti and 200–300 V for Ti-40Nb substrates.

**Figure 2:**
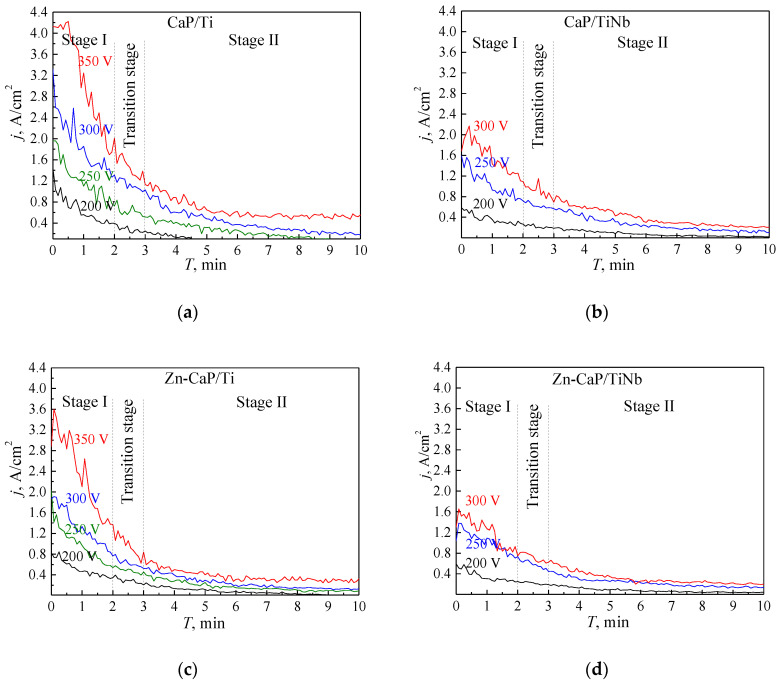
Plots of the current density vs the MAO time for the deposition of the coatings under different applied voltages: (**a**) CaP coating on Ti; (**b**) Zn-CaP coatings on Ti-40Nb; (**c**) Zn-CaP coating on Ti; (**d**) Zn-CaP coating on Ti-40Nb.

In Figure 2 the graphs of the current density versus the MAO processing time for the deposition of CaP-based and modified CaP (e.g., Zn-CaP) coatings on both substrates under the different applied voltages is represented. The presence of fluctuations for all the curves points arises due to the pulsed nature of micro-arc discharges, causing transport of electrolyte substance inside the discharge channels, and its subsequent deposition in the form of a coating. During the MAO process, the current density monotonously decreases, which is caused by the change of the layer structure in the metal-electrolyte interface. This change is due to the formation of the oxide layer and subsequent formation of the dielectric CaP layer on the sample surface. An increase in the voltage leads to the increment of current density during the formation of all four types of coatings. It is attributed to the increment of micro-arc discharges’ intensity leading to the acceleration of the coatings’ growth.

The coating deposition during the MAO synthesis on both types of substrates can be divided into two main stages. In the first stage I (up to ~3 min), the current density intensively decreases. It corresponds to the high rate of the coating growth under the impact of numerous intensive micro-arc discharges occurring as a result of a localized electric breakdown of the coating. In the second stage II (from 3 to 10 min) the current density is minimal, reaching a plateau. In this case, the formed dielectric CaP coating has sufficient thickness that counteracts the electrical breakdowns. Therefore, the coating’s growth decelerates. The time limits of the above-mentioned stages are not universal and could shift in response to the substrate material, the elemental composition of electrolyte, electro-physical parameters of the process being used. Therefore, we propose to introduce a transition zone (2–3 min) between the stage I and stage II of the deposition process. The duration of a transition zone could act like a damper area between the two stages that would take into account the synthesis conditions of the coatings and change accordingly.

It should be noted that the MAO processing of the coating deposition on Ti is characterized by a higher current density (Figure 2a,c) than that for the Ti-40Nb (Figure 2b,d). We associate it with the electro-physical, thermal, and thermodynamical differences between the Ti and Nb as well as their oxides (TiO_2_, Nb_2_O_5_). The Nb has a higher thermal conductivity at 300 K (54.5 W/m·K), and lower electrical resistivity (0.15 μΩ·m) than Ti which thermal conductivity is 15.5 W/m·K, and electrical resistivity is 0.55 μΩ·m) [39]. Therefore, the MAO processing occurs more intensively on the Ti-40Nb substrate than on Ti, possibly, leading to a higher rate of the coating growth. Also, the current densities during the MAO processes for deposition of the CaP coatings on both substrates are significantly higher (Figure 2a,b) than those for deposition of the modified Zn-CaP or Cu-CaP coatings (Figure 2c,d). It can be attributed to the presence of the electro-conductive Zn^2+^ or Cu^2+^ ions formed in the thermo-chemical reactions during the process. In this case, the reactive capacity of the electrolyte components increases; therefore, the coating deposition rate increases as well.

### 3.2. Morphology and Topography Characterization

**Figure 3:**
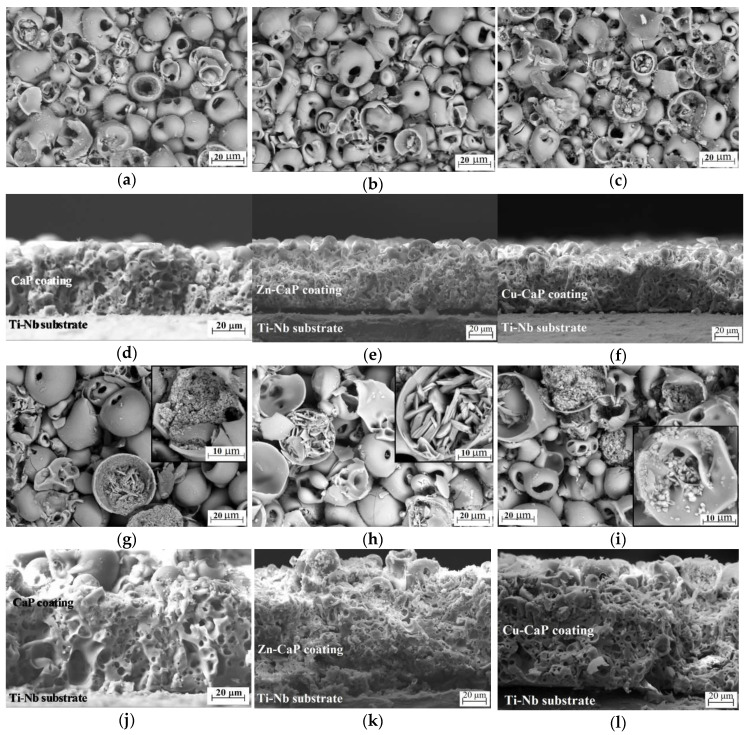
SEM images of the surface and cross-section of the coatings deposited on Ti-40Nb alloy at voltages of 200 V (**a**–**f**) and 300 V (**g**–**l**): (**a**,**d**,**g**,**j**) CaP coating; (**b**,**e**,**h**,**k**) Zn-CaP coating; (**c**,**f**,**i**,**l**) Cu-CaP coating.

The SEM studies revealed that all coating types on both Ti and Ti-40Nb substrates have similar surface morphology and the bulk structure. The surface morphology of the coatings is represented by the spheroidal elements (spheres and hemispheres) with the characteristic open pores and local particulates (Figure 3a–c,g–i). 

The observed morphology is similar to the structure with a well-known mechanism of the formation and collapse of the vapor-gas and plasma bubbles in the channels of micro-arc discharges described in the work [40,41,42]. The occurrence of micro-discharge during MAO is due to the formation of a vapor-gas bubble pore due to the heating of the oxide layer at the substrate’s interface. After an electric breakdown, the vapor-gas bubble transforms into a plasma bubble with a simultaneous formation of a discharge channel (pore) as a natural to keep away the heated electrolyte substance. The bubble expands along the pore until it reaches the pore mouth. The decay of the micro-arc leads to the rapid cooling and collapse of the vapor-gas bubble. The bubbles cool down to form the spheres and the products of this reaction condense at the bottom and on the walls of the channel that were formed under the action of micro-discharge. Upon crystallization, the channel is blocked by the products of electrochemical and plasma-chemical reactions. Possible electrochemical reactions at the metal-electrolyte interface for the Ti substrate were described earlier in [43].

The SEM analysis of the cross-section of the coating revealed that the structural elements could be found only on the coating surface. It became evident that the coatings have a complex porous structure that continues through its thickness formed by multiple branched rounds, elliptic, and isometric pores. The pore sizes vary in the range of 0.5–15 µm for the coatings produced at low voltages of 200–250 V (Figure 3d–f).

An increase in the applied voltage from 200 to 350 V leads to a rise in the intensity of the micro-arc discharges, and as a result, the sizes of discharge channels start to grow, followed by the increasing temperature. It resulted in a partial transformation of spheres to hemispheres inside, which the new plate-like crystals with sizes up to 15 µm were formed (Figure 3g–i). For the coatings deposited at 200–250 V on both types of substrates, the dimensions of the spheres and pores measured according to the secant method were found to be 4–30 µm, and 0.5–15 µm, respectively. At the same time, for the coatings deposited at 300–350 V on both substrates, the sizes of the spheres and pores were 10–40 µm, and 0.5–30 µm, respectively. As the MAO voltage increases, the average size of the spheres and pores in all the types of the coatings on both substrates increased in the ranges of 20–26 µm, and 4–8 µm, respectively. It should be noted that, in addition to the microsized pores detected inside, the coatings close to the substrate area showed large pores (macropores) with sizes of 15–30 µm (Figure 3j–l). It is possible that these macropores are formed under the action of high voltages (300–350 V), generating “cascades” of high-intensity current pulses in the limited areas. The emergence of such cascades is typical for the initial stage of the MAO process (stage I in Figure 2). Therefore, macropores are localized mainly in the lower layers of the coatings. Dunleavy et al. [44] also reported the explanations and characterization of such discharge cascades during the MAO processing.

**Figure 4:**
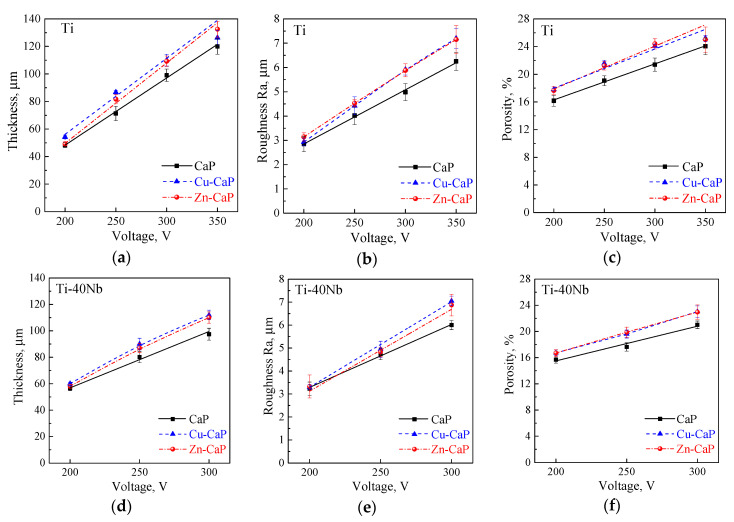
Plots of the thickness (**a**,**d**), surface roughness (**b**,**e**), and porosity (**c**,**f**) of all the types of the coatings on both Ti (**a**–**c**) and Ti-40Nb (**d**–**f**) substrates against the MAO applied voltage.

Figure 4 shows that the increase in the MAO voltage leads to a linear increase in the thickness, surface roughness, and porosity of all types of coatings on both Ti and Ti-40Nb substrates. It can be seen that the coatings, modified with Zn or Cu on both substrates, are characterized by the larger thickness, surface roughness, and porosity than the CaP coatings deposited at the same electrical voltages. As the applied voltage increases, the thickness and roughness of the Zn-CaP and Cu-CaP coatings grow more intensive than that of the CaP coatings. This is evident by a small difference of corresponding functions’ slope angles (Figure 4a,b,d,e). As it was mentioned previously, this is due to the presence of conducting ions (Zn^2+^ or Cu^2+^) during the coating formation. For the coatings on Ti, the increase of the voltage from 200 to 350 V leads to the increase in the thickness, surface roughness, and porosity in the ranges of 45–130 µm, 2.7–7.0 µm, and 16–25%, respectively. At the same time for the coatings on Ti-40Nb, the increase of the voltage from 200 to 300 V leads to the rise of the thickness, surface roughness and porosity in the ranges of 57–100 µm, 3.2–7 µm, and 15–23%, respectively.

The surface topography substantially affects the biological response to the material. It has been established that the cells’ alignment and migration, together with the cellular production of organized cytoskeletal arrangements and other sorts of biological responses, are significantly influenced by the surface roughness [45]. Some reports support the fact that certain surface roughness, at the nano- and micro-metric scales, can lead to successful osseointegration of medical implants [46,47]. The proliferation, differentiation, and matrix production of osteoblastic cells, as well as the production of local growth factors and cytokines, are influenced by the surface roughness. However, the question of roughness values, required for the surface of the biomaterial, is still under active discussion. Sammons et al. [48] noted that the surface roughness could be varied in the full range from nano- to microscopic scales positively impact the biological interaction between the implant and bone tissue, as it has the same order of values as cells and large biomolecules. At the same time, in [49] it has been shown that microporous biocoatings’ surfaces with the roughness of 2.5 µm < *Ra* < 5.0 µm promote successful stromal stem cell’s adhesion, proliferation, and differentiation into the bone tissue. However, in [50], the authors point out that the surface of implants with nano-roughness is more applicable as it promotes the adhesion of osteoblastic cells, intensifies cells’ functions (alkaline phosphatase synthesis, calcium deposition, and collagen secretion) and prevents the growth of “competitive” fibroblast cells.

**Figure 5:**
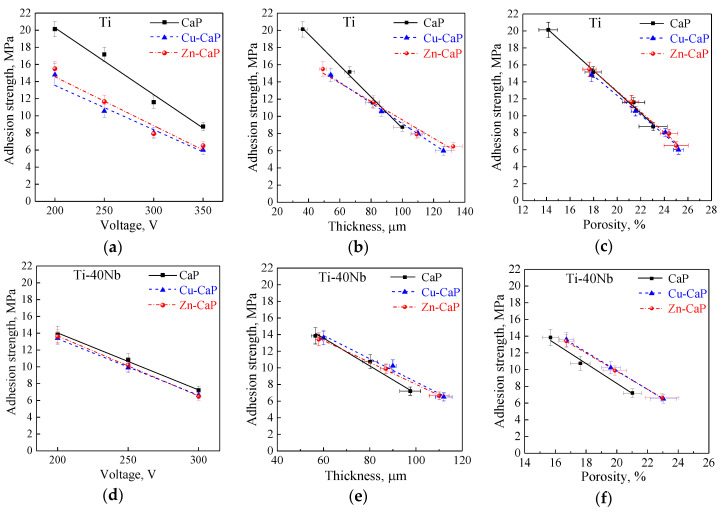
Plots of the adhesion strength of all the types of coatings on both Ti (**a**–**c**) and Ti-40Nb (**d**–**f**) substrates against the applied voltage (**a,d**), the coating thickness (**b,e**) and surface porosity (**c,f**).

It is well-known that the critical service property of the coating is a value of the coating to substrate adhesion. It was revealed that the adhesion of all types of coatings on both substrates decreases linearly with an increase in the MAO voltage (Figure 5). 

Most certainly, adhesion strength decrease is due to the increase of the thickness and porosity (see Figure 4a,c,d,f), which leads to an increase in the value of internal stresses in the coatings. It should be noted that the presence of local “macro-pores” in the coating’s inner layer (see Figure 3j–l) can also significantly decrease coating’s strength properties and, in the first place, adhesion strength. Figure 5 demonstrates an increase in the voltage in the range of 200–350 V and, consequently, an increase in the coating thickness and porosity, the coating adhesion strength to both substrates decreases linearly. The adhesion strength to the Ti substrate decreases from 19 to 8 MPa for the CaP coatings and from 16 to 5 MPa for the modified Zn-CaP and Cu-CaP coatings (Figure 5a–c). At the same time, the coatings on Ti-40Nb are characterized by the low adhesion, which decreases from 14 to 6 MPa (Figure 5d–f). It can be seen that the coatings formed on the Ti-40Nb have lower adhesion strength than those on the Ti substrates. It can be due to the larger thickness and pore sizes of the coatings on the Ti-40Nb alloy compared to the coatings on Ti.

According to the International Standard ISO 13779-2:2018 [51], the coating adhesion strength for medical devices must not be smaller than 15 MPa. In our case, all types of coatings deposited at more than 200 V on both substrates are found to possess lower than required adhesion strength, which is insufficient for medical applications. In this regard, to improve the coating adhesion, the surface of the substrates could be preliminarily sandblasted with subsequent chemical etching. The treatment allows improving the adhesion strength up to 25 MPa for all types of coatings deposited at low voltages of 200–250 V on both types of substrates, as described in detail in our previous report [39].

### 3.3. Phase, Elemental and Chemical Composition

**Figure 6:**
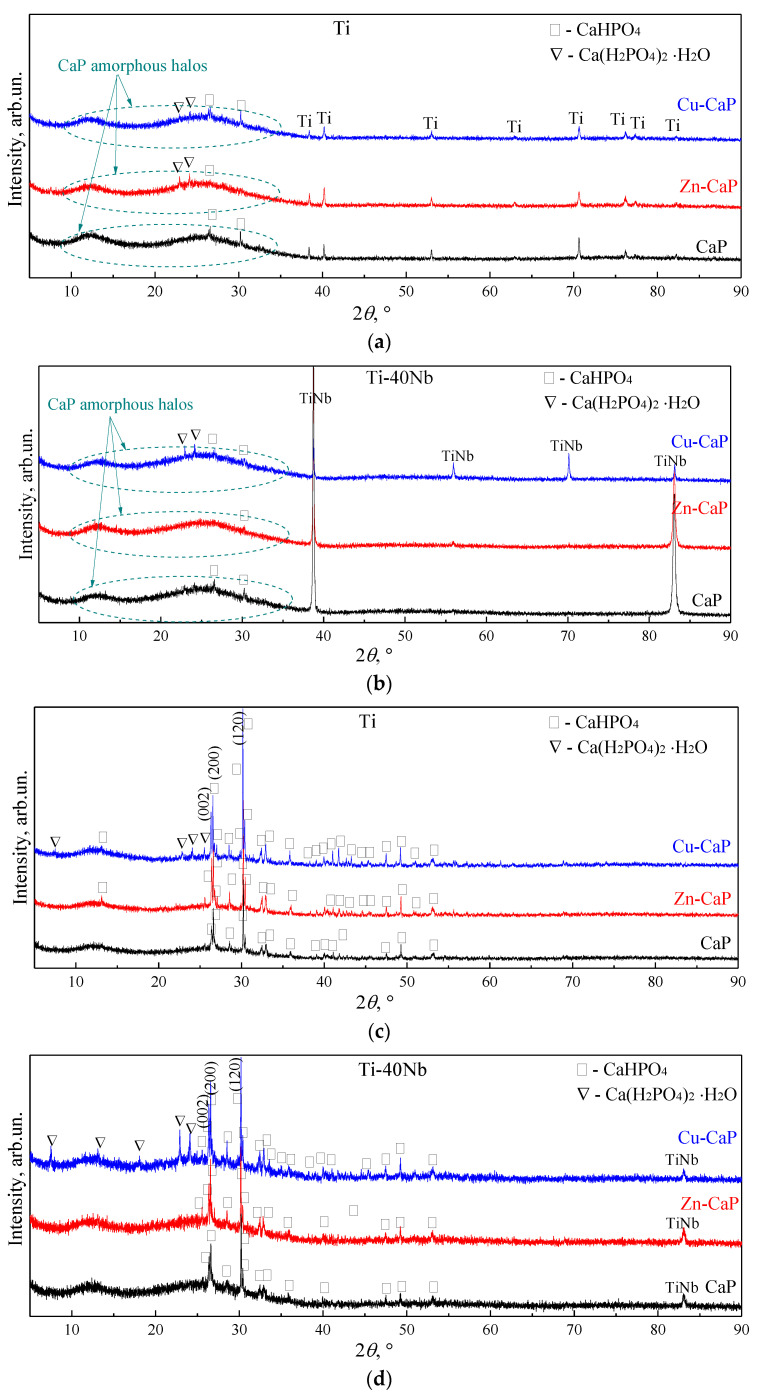
XRD patterns of all the types of coatings deposited at applied voltages of 200 V (**a**,**b**), 350 V (**c**) and 300 V (**d**) on Ti (**a**,**c**) and Ti-40Nb (**b**,**d**) substrates.

XRD studies revealed that the Zn-CaP, Cu-CaP, and CaP coatings deposited at low voltages of 200–250 V on both Ti and Ti-40Nb substrates are found to be mainly in the X-ray amorphous state. It is evident due to the appearance of two diffusive halos in the range of 2*θ* angles from 10° to 35° in the XRD patterns (Figure 6a,b). 

Also, these XRD patterns include the weak reflection peaks of the hexagonal α-phase Ti (ICDD #44-1294) and high-intensity reflection peaks of the cubic β-phase TiNb (ICDD #89-4913), corresponding to the substrate materials. In this case, the weak reflection peaks from the dicalcium phosphate anhydrous (CaHPO_4_, DCPA, monetite mineral, ICDD #70-0359) are observed in the XRD patterns for all types of coatings deposited at low voltages of 200–250 V on both substrates. In addition to the DCPA phase, the weak reflection peaks of monocalcium phosphate monohydrate (Ca(H_2_PO_4_)_2_·H_2_O, MCPM, ICDD #70-0090) phase are observed in the XRD patterns for modified Zn-CaP and Cu-CaP coatings on both types of substrates.

As the applied voltage increases from 250 to 350 V, the number and intensity of the reflections from different crystallographic planes of the DCPA phase increased significantly (Figure 6c,d). These reflections are more intensive for the coatings on Ti substrate than that for the coatings on Ti-40Nb. In addition, these reflections are more intensive for the modified Zn-CaP and Cu-CaP coatings than for the CaP coatings on both substrates. The weak reflections from the second MCPM phase are observed in the XRD patterns of the modified Zn-CaP and Cu-CaP coatings deposited at high voltages (300–300 V). Therefore, it could be concluded that the crystallinity of the formed coatings increases in the following order: CaP/TiNb < Zn-CaP/TiNb < Cu-CaP/TiNb < CaP/Ti < Zn-CaP/Ti < Cu-CaP/Ti.

Rietveld refinement confirmed that the DCPA and MCPM phases in all types of coatings deposited at different applied voltages on both substrates have similar triclinic crystalline structure with unit cell parameters close to those for the reference phase (Table 2). For all types of deposited coatings, DCPA phase is mainly oriented in (120) and (200) planes, which are evident in the corresponding XRD patterns (Figure 6c,d). Different crystallographic planes have their own intrinsic charge, due to this selective adsorption of various ions and organic compounds could occur. The abovementioned planes which are energetically favorable planes for DCPA have a negative charge due to OH– groups and according to [52] may promote HA crystallization. The increase of the MAO voltage from 200 to 350 V leads to the rise of the DCPA crystallite size from 71 to 227 nm for all types of coatings. For the second MCPM phase, no regularities of crystallite size change depending on the coating type and the applied voltage were found.

The formation of DCPA and MCPM phases in the coatings can be associated with the recrystallization of the previously amorphous CaP phase due to the growth of temperature inside the micro-arc discharges with an increase in the applied voltage. Previously [38], we described the mechanism of the formation of the DCPA and β-calcium pyrophosphate (β-CPP, β-Ca_2_P_2_O_7_) phases in the micro-arc lanthanum-silicon-incorporated CaP coatings. Firstly, the main electrolyte components, which are phosphoric acid and calcium carbonate, react to form an MCPM and dicalcium phosphate dihydrate (CaHPO_4_·H_2_O, brushite mineral) [43]. During the MAO processing under high voltages (300–350 V), the brushite transforms to the monetite at the temperatures above 135 °C and the amorphous CPP at the temperatures above 400 °C. After that, with increasing temperature above 530 °C the polymorphic transformations from amorphous CPP to γ-, β- and α-CPP modifications occur according to the following reaction [53]:

(1) $$\begin{array}{l} {\mathrm{CaHPO}}_{4}\cdot {2H}_{2}O\overset{135^{\circ }C}{\to }{\mathrm{CaHPO}}_{4}\overset{360-450^{\circ }C}{\to }\mathrm{amorphous}-{\mathrm{Ca}}_{2}P_{2}O_{7}\overset{530^{\circ }C}{\to }\\ \gamma -{\mathrm{Ca}}_{2}P_{2}O_{7}\overset{750^{\circ }C}{\to }\beta -{\mathrm{Ca}}_{2}P_{2}O_{7}\overset{1171-1191^{\circ }C}{\to }\alpha -{\mathrm{Ca}}_{2}P_{2}O_{7} \end{array}$$

Therefore, the increase in the MAO voltage leads to a coating structure transformation from X-ray amorphous to the amorphous-crystalline state. These XRD data are in agreement with the SEM results, indicating the incorporation of plate-shaped crystals on the surface of the coatings, formed at high voltages (Figure 3g–i). The formed crystals are resembling the shape of the crystalline DCPA phase [54].

The presence of CaHPO_4_ phase in the coating is of particular interest due to the chemical agreement of this phase with the bone matrix [54]. The coatings containing monetite at pH ≈ 7 are more soluble than HA-based materials [55]. Moreover, monetite, as well as other acidic CaPs has high osteoinductive properties, i.e., stimulates osteogenesis. During the dissolution, there is a local increase in pH medium in the implant-bone interface that partially dissolves bone apatite, and induce desorption of specific osteoinductive BMP-type proteins from its surface [56]. Therefore, monetite is soluble in the body fluids and also promotes nucleation and growth of bone apatite during the biomineralization. 

The quantitative EDX microanalysis revealed the following elemental composition of the all types of the coatings deposited at the different applied voltage on Ti: Са (4.9–11.4 at%), Р (14.3–21.1 at%), О (52.0–73.4 at%), Ti (8.1–17.8 at%), Zn, or Cu (≤0.4 at%). At the same time, the coatings on Ti-40Nb have following elemental composition: Са (5.7–9.8 at%), Р (15.8–25.2 at%), О (51.2–70.5 at%), Ti (5.8–12.2 at%), Nb (3.4–7.9 at%), Zn or Cu (≤0.3 at%). As the applied voltage increased from 200 to 350 V, there was an increase in the Ca and O concentrations when Ti, Nb, and P concentrations decrease. The EDX allowed us to detect the presence of Zn and Cu, but not quantitatively assess their concentrations. At low MAO voltages, firstly negatively charged phosphate-ions in the electrolyte are deposited on the positively charged specimen (anode). As the applied voltage increases, it leads to an incremental increase in micro-arc discharges’ intensity and elevated electrolyte heating. As a result, the reaction capacity of all the electrolyte components is increased, and positively charged calcium ions and calcium-incorporated compounds are also deposited. Moreover, with the voltage increase, the coatings’ thickness increases too, and hence, the observed by EDX concentration of substrate elements (Ti and Nb) in the coatings decreases.

**Figure 7:**
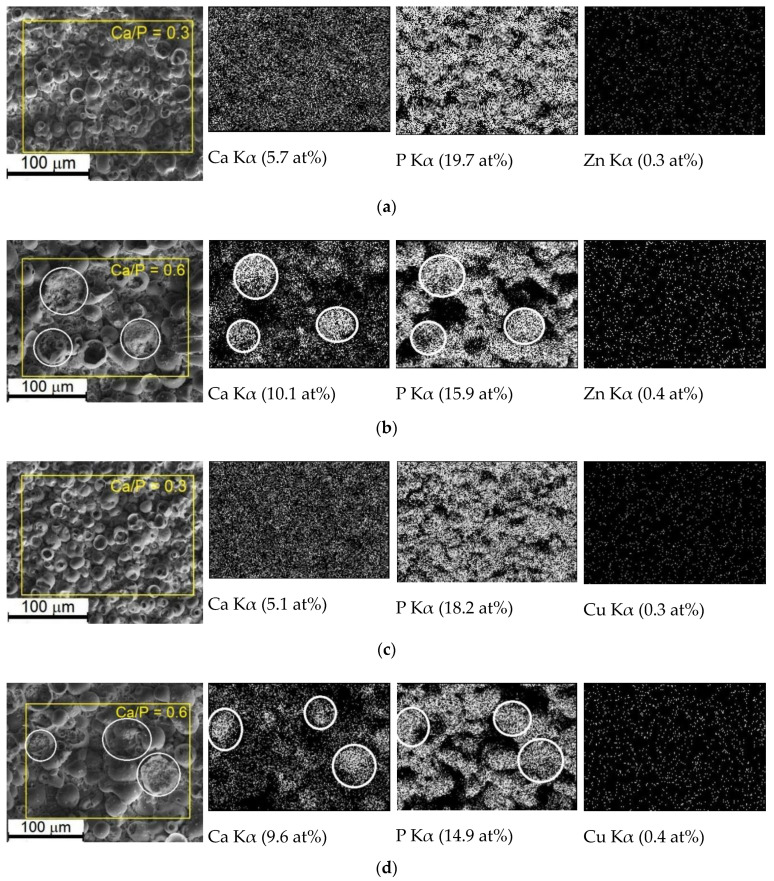
Typical SEM images and EDX grey-level maps of the Ca, P, Zn and Cu concentrations (marked with white color) in the Zn-CaP (**a**,**b**) and Cu-CaP (**c**,**d**) coatings deposited on Ti at applied voltages of 200 V (**a**,**c**) and 300 V (**b**,**d**).

Figure 7 shows the EDX grey-level mapping of the Ca, P, Zn and Cu elements distribution in the Zn-CaP and Cu-CaP coatings deposited on Ti substrate at 200 and 300 V. For the coatings deposited at low voltages of 200–250 V on both substrates, it is shown that all the elements are distributed homogeneously (Figure 6a,c). These coatings are characterized by a low Ca/P atomic ratio of 0.3. With increasing MAO voltage, the Ca concentration increases, and P concentration decreased (Figure 7b,d). In this case, Ca and P are mainly localized in the regions of the plate-like crystals accumulation. Increasing the calcium concentration leads to the increment of Ca/P atomic ratio from 0.3 to 0.7 in the coatings on Ti and from 0.2 to 0.5 in the coatings on Ti-40Nb alloy.

Relatively low Ca/P ratio of less than 1.0 in all the deposited coatings (for stoichiometric HA, Ca/P = 1.67; for stoichiometric CaHPO_4_, Ca/P = 1.0) could be explained by the rough surface leading to underestimated values and fact that alongside with crystalline phases of monetite and β-calcium pyrophosphate the amorphous CaP substance with cations of Ti and Nb is abundant in the coating. Amorphous CaP compounds are characterized by Ca/P atomic ratio of 0.3–0.7 [32].

**Figure 8:**
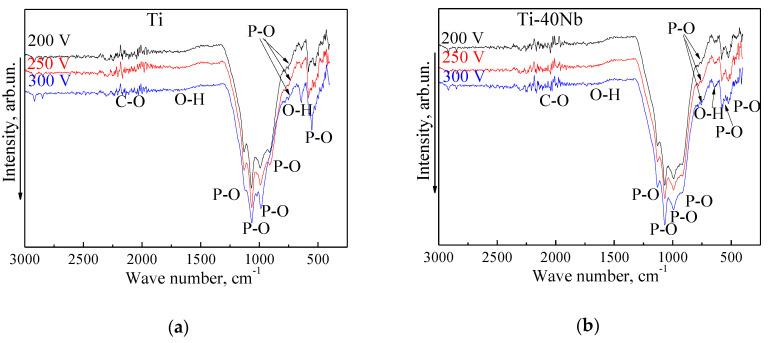
Typical FT-IR spectra of the MAO coatings deposited at different voltages on both Ti (**a**) and Ti-40Nb (**b**) substrates.

The FT-IRS studies of the MAO coatings deposited on both Ti and Ti-40Nb substrates at different voltages are shown in Figure 8. The FT-IR spectra of all types of coatings include the intensive adsorption bands from the asymmetric and symmetric vibrations of the P–O phosphate bond with the maximum absorption in the region of 930–1130 cm^−1^. A broadened blurred absorption band of OH– groups associated with water adsorbed from the air is observed in FT-IR spectra at 1620–1650 cm^−1^. A shoulder in the region of 730–800 cm^−1^ corresponds to the vibrations of P–O–P phosphate bridge bonds. Sufficiently intensive adsorption bands at 520–600 cm^−1^ indicate the presence of triply degenerated deformation vibrations of О–Р–О phosphate bonds. The adsorption bands at 630–660 cm^−1^ are attributed to the O–H bonds of acid phosphate, such as НРО_4_-groups (CaHPO_4_) [57]. The results of FT-IR are in good correlation with the XRD data described above. The results show strong P–O phosphate and O–H bonds in both amorphous and crystalline phases of the coatings.

### 3.4. Microstructure Characterization

**Figure 9:**
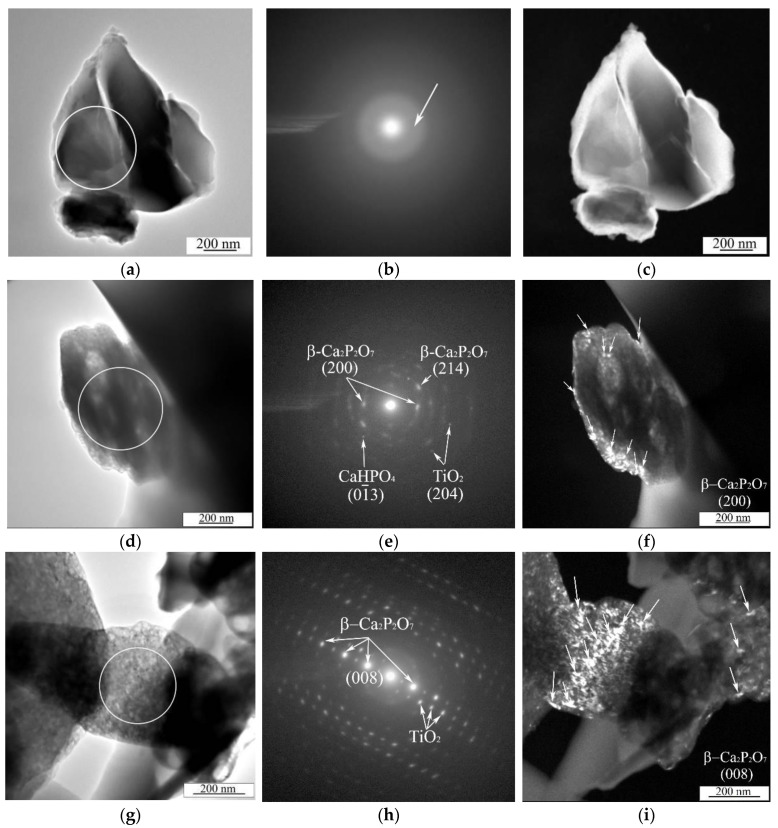
BF TEM (**a**,**d**,**g**) and DF TEM (**c**,**f**,**i**) images and SAED patterns (**b,e**,**h**) of the particles of the CaP (**a**–**c**), Cu-CaP (**d**–**f**) and Zn-CaP (**e**–**g**) coatings deposited on Ti at 300 V. The SAED patterns were observed within the regions of interest that are highlighted in BF TEM images.

In Figure 9, bright field (BF) and dark field (DF) TEM images with SAED patterns for fragments of the MAO coatings are shown. The TEM studies revealed that the CaP coatings without modifications on both Ti and Ti-40Nb substrates at different voltages have an amorphous microstructure (Figure 9a–c). It is confirmed by the SAED pattern with diffusive halos (Figure 9b) and DF TEM image without any visible crystallites (Figure 9c). In contrast to the CaP coatings, the Zn CaP and Cu CaP coatings, deposited on both types of substrates at different voltages, have an amorphous crystalline microstructure (Figure 9d–i). Both diffused halos and numerous ring-reflexes or point reflexes from different phases were observed in SAED patterns for the Cu CaP (Figure 9e), or the Zn CaP coatings (Figure 9h), respectively. The interpretation of the SAED patterns of the coatings on Ti illustrates the complex polyphase composition with the following crystalline phases: β Ca2P2O7 with tetragonal lattice (β CPP, ICDD #09 0346), CaHPO4 with triclinic lattice (DCPA, ICDD #70 0359), TiO2 (anatase) with tetragonal lattice (ICDD #21 1272). The numerous crystallites in (200) and (008) reflections of the β Ca2P2O7 phase are observed in the BF TEM images (Figure 9e,f,h,i). These crystallites have a size of 10 to 80 nm and comprised of equiaxial shape. The SAED analysis of the coatings on the Ti 40Nb alloy indexed the same phases and additional phase of Nb2O5 with monoclinic lattice (ICDD #37 1468). The nanocrystalline DCPA phase detected in TEM was identified earlier by the XRD method. Large crystals up to 15 µm in size observed in SEM images (Figure 3g–i) are believed to be DCPA phase.

Moreover, the nanocrystalline β-CPP phase was identified using TEM for samples deposited at high applied voltages (300–350 V). The formation of this phase in the nanocrystalline state could be due to local high-temperature processes in electrolyte associated with electrical breakdown. According to the polymorphic transformation scheme (Equation (1)), β-CPP phase is formed at the temperatures above 750 °С. Earlier in the paper [39] it has been illustrated that the oxides of titanium and niobium are typically localized in the interface layer between the metal substrate and the coating. In our case, due to the sample preparation for TEM investigation, both particles from the upper layer of the coatings and the ones that were close to the metallic substrate could appear in the replica. Therefore, when the SAED patterns were analyzed, we found both the phases related to calcium phosphates and phases related to titanium oxides (TiO_2_ anatase) and niobium oxides (Nb_2_O_5_).

## 4. Conclusions

The studies of the microstructure, composition, and properties of the CaP and modified Zn-CaP or Cu-CaP coatings prepared by MAO on pure Ti and Ti-40Nb alloy allows to draw the following conclusions.

1. The CaP coatings without modifications deposited on both Ti and Ti-40Nb substrates at different voltages had mainly an amorphous microstructure with a minimal content of CaHPO_4_ phase. At the same time, the modified Zn-CaP and Cu-CaP coatings that were formed on both types of the substrates at different voltages had an amorphous-crystalline structure, containing micro-sized CaHPO_4_ and Ca(H_2_PO_4_)_2_·H_2_O phases, and nano-sized β-Ca_2_P_2_O_7_, CaHPO_4_, TiO_2_, and Nb_2_O_5_ phases.

2. Depending on the crystallinity degree, the coatings could be arranged in the following order: CaP/TiNb < Zn-CaP/TiNb < Cu-CaP/TiNb < CaP/Ti < Zn-CaP/Ti < Cu-CaP/Ti.

3. The increase in the applied voltage led to a linear increase in the thickness, roughness, and porosity of the MAO coatings on the Ti and Ti-40Nb substrates, and a linear decrease of their adhesive strength values.

4. The increase in the voltage did not affect the Zn or Cu concentration which did not exceed 0.4 at% and led to an increase in the Ca/P atomic ratio from 0.3 to 0.7.

5. The applied voltage in the range of 200–250 V provided to form the MAO coatings on the Ti and Ti-40Nb alloy with a required combination of properties that allow using those coatings for further biological tests: Ca/P atomic ratio of at least 0.5; the thickness of 35–55 µm; the roughness *Ra* of 2–5 µm; the surface porosity of 15–22%; the adhesion strength more than 15 MPa.

## Figures and Tables

**Table 1:** The studied materials and the MAO parameters.

Materials	
Material Substrate	1. Ti 2. Ti-40Nb
Electrolyte Composition	1. Ca_10_(PO_4_)_6_(OH)_2_, CaCO_3_, H_3_PO_4_, H_2_O (CaP Coating) 2. Ca_9.9_Zn_0.1_(PO_4_)_6_(OH)_2_, CaCO_3_, H_3_PO_4_, H_2_O (Zn-CaP Coating) 3. Ca_9.9_Cu_0.1_(PO_4_)_6_(OH)_2_, CaCO_3_, H_3_PO_4_, H_2_O (Cu-CaP Coating)
**MAO Parameters**	
Frequency, Hz	50
Pulse duration, µs	100
Time, min	10
Voltage, V	200, 250, 300, 350

**Table 2:** Unit cell parameters and crystallite size of the DCPA and MCPM phases in the coatings.

Type of the Coating	Voltage, V	DCPA Cell Parameters (ICDD #70-0359)				MCPM Cell Parameters (ICDD #70-0090)			
		a, Å	b, Å	c, Å	Crystallite Size, nm	a, Å	b, Å	c, Å	Crystallite Size, nm
Reference Phase	–	6.910	6.627	6.998	–	5.626	11.889	6.473	–
CaP/Ti	200	6.921	6.650	7.015	71	–	–	–	–
Zn-CaP/Ti		6.910	6.640	6.990	100	5.626	11.899	6.475	75
Cu-CaP/Ti		6.905	6.635	6.996	96	5.630	11.900	6.470	108
CaP/TiNb	200	6.918	6.647	7.001	150	–	–	–	–
Zn-CaP/TiNb		6.899	6.636	6.997	–	–	–	–	–
Cu-CaP/TiNb		6.909	6.629	6.989	100	5.624	11.899	6.473	134
CaP/Ti	350	6.889	6.628	6.985	172	–	–	–	–
Zn-CaP/Ti		6.900	6.636	6.994	227	–	–	–	–
Cu-CaP/Ti		6.897	6.636	6.997	186	5.623	11.919	6.481	76
CaP/TiNb	300	6.896	6.632	6.994	150	–	–	–	–
Zn-CaP/TiNb		6.895	6.629	6.985	212	–	–	–	–
Cu-CaP/TiNb		6.901	6.637	6.997	170	5.628	11.897	6.479	89
